# Influence of the Tibial Tunnel Angle and Posterior Tibial Slope on “Killer Turn” during Posterior Cruciate Ligament Reconstruction: A Three-Dimensional Finite Element Analysis

**DOI:** 10.3390/jcm12030805

**Published:** 2023-01-19

**Authors:** Fan Yang, Takuji Yokoe, Koki Ouchi, Takuya Tajima, Etsuo Chosa

**Affiliations:** Division of Orthopaedic Surgery, Department of Medicine of Sensory and Motor Organs, Faculty of Medicine, University of Miyazaki, Miyazaki 889-1692, Japan

**Keywords:** posterior cruciate ligament reconstruction, killer turn, posterior tibial slope, tibial tunnel angle, anterior opening wedge high tibial osteotomy, finite element analysis

## Abstract

This study aimed to evaluate the influence of various posterior tibial slopes (PTSs) and tibial tunnel angles (TTAs) on “killer turn” in posterior cruciate ligament (PCL) reconstruction by using three-dimensional finite element analysis (FEA). The study models were created using computed tomography images of a healthy young Asian male. Using SolidWorks, PCL grafts and tibial bone tunnels at different tibial drilling angles (30°, 45°, 60°) were developed. Anterior opening wedge high tibial osteotomy (aOW-HTO) was performed to evaluate the influence of the PTS (+8°, +4°, native, −4°, −8°). An FEA was performed utilizing the ANSYS software program. In the same PTS model, the peak of the equivalent Von Mises stress in PCL grafts decreased as the angle of the TTA increased. In the same TTA model, the peak of the Von Mises in PCL grafts decreased as the PTS angle increased. The “high-contact stress area” (contact stress greater than 10 MPa) was diminished when the TTA and PTS were increased. aOW-HTO was used to steepen the PTS, and a larger TTA may reduce the stress at the “killer turn” during PCL reconstruction. In conclusion, the study findings suggest that using aOW-HTO to steepen the PTS and a larger TTA may reduce the stress at the “killer turn” during PCL reconstruction. The usefulness and safety of this surgical procedure need to be evaluated in future clinical studies.

## 1. Introduction

The posterior cruciate ligament (PCL) is principally responsible for limiting the posterior translation and external rotation of the knee joint [1]. It was reported that, if PCL injury was left untreated, 33.7% of patients developed medial femoral condyle osteoarthritis, and 46.7% developed patellar osteoarthritis [2,3]. Arthroscopic PCL reconstruction has been reported to be an effective treatment for both acute and chronic PCL injuries [4]. Complications after PCL reconstruction have been reported to occur in up to 53% of patients [5]. Among the common postoperative complications are graft failure with recurrent pain and instability [5], with nearly 30% of patients suffering from a failure that requires revision PCL reconstruction [6]. The most prevalent causes of reconstruction failure are posterolateral corner deficiency (40–77%) and femoral or tibial tunnel misplacement (30–41%) [6,7]. Therefore, investigation regarding risk factors for failure of PCL reconstruction and advancements in surgical procedures will be needed.

Because of the intricate spatial architecture of the knee joint, PCL grafts at the exit of the tibial tunnel are susceptible to the “killer turn” effect [8,9,10]. The “killer turn” is not only a main cause of the wear and retear of the PCL graft but also a risk factor for enlargement of the tibial tunnel after PCL reconstruction [10,11]. Li et al. reported that the enlarged tibial tunnel as well as repetitive friction and wearing of the PCL graft at the “killer turn” may be associated with residual laxity after PCL reconstruction [12]. To eliminate the “killer turn” effect, tibial tunnel creation using a larger tibial tunnel angle (TTA) [13] or an anterolateral portal [14], lower tibial tunnel placement [15], and smoothing the posterior tibial facet [16] have been proposed. Theoretically, as the TTA increases, the “killer turn” effect decreases [13]. However, the TTA has anatomical limitations [17]. If the TTA is too large, the posterior wall of the tibial tunnel will blow out.

Patient-specific bone morphologies have recently received focus in studies regarding PCL reconstruction. The width of the intercondylar notch, the shape and size of the tibial eminence, the posterior tibial slope (PTS), and knee malalignment have been reported to be risk factors for both PCL injury or failure after PCL reconstruction [6,18,19]. Among these morphological factors, many studies have focused on the influence of PTS on the force in the PCL graft. Clinical studies have reported that a flat PTS may be associated with an increased risk of graft failure after PCL reconstruction [2,20,21,22]. However, biomechanical studies investigating the role of PTS in PCL reconstruction are lacking. Additionally, several researchers have focused on the application of high tibial osteotomy (HTO) to correct the PTS angle during anterior cruciate ligament (ACL) and PCL reconstruction [23,24,25]. Anterior opening wedge high tibial osteotomy (aOW-HTO) may serve as a promising surgical option for adjusting the PTS [22,26]. However, from a biomechanical perspective, the application of aOW-HTO to mitigate the “killer turn” effect has not been studied well.

To our knowledge, no study has evaluated the influence of the combination of the PTS and TTA on the stress distribution in the “killer turn” of the PCL graft. The present study therefore investigated whether or not different TTAs and PTSs in PCL reconstruction demonstrated an advantage in attenuating the “killer turn” effect using a three-dimensional (3D) finite element analysis (FEA). We hypothesized that a large TTA and steep PTS in PCL reconstruction would effectively alleviate the “killer turn” effect.

## 2. Methods

This study was approved by the institutional review board (Approval No. O-0994). All procedures were in accordance with the ethical standards of the responsible committee on human experimentation and with the 1975 Declaration of Helsinki, as revised in 2013. Written informed consent was obtained from the subject.

### 2.1. Data Acquisition

We recruited a healthy 30-year-old male volunteer (height: 180 cm, weight: 90 kg). Computed tomography (CT) images were obtained from the subject using high-resolution CT apparatus (Canon Aquilion One, Tochigi, Japan). The scans of the knee joint at 90° flexion were obtained in the lateral position with a slice distance of 0.5 mm and a field of view of 500 mm. During PCL reconstruction, the stress on the graft is highest when the knee is flexed at 90° [27].

### 2.2. 3D Reconstruction of the Knee Joint and FEA Modeling

**Figure 1:**
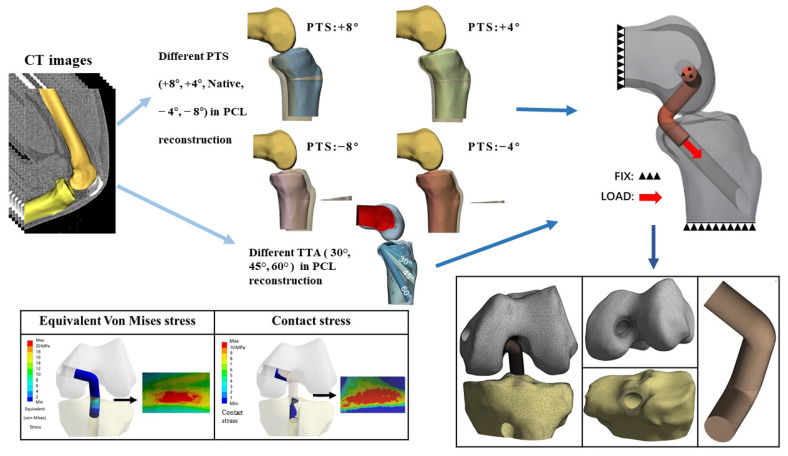
Flowchart of the study model development. TTA: tibial tunnel angle, PTS: posterior tibial slope.

The MIMICS software program (ver. 24.0; Materialise, Leuven, Belgium) was used to reconstruct a 3D model of the knee joint. Segmentation using MIMICS was performed by an experienced orthopedic surgeon. The contours of the femur and tibia were segmented from the CT images. These parts of the knee model were exported as stereolithography (STL) files and imported to the Materialise 3-Matic software program (ver. 16.0; Materialize) to smooth the models. We simulated surgery on the model using the computer-aided design (CAD) software program SolidWorks 2015 (Dassault Systemes Inc., Paris, France). PCL grafts and tibial tunnels with different TTAs (30°, 45°, 60°) were developed. aOW-HTO was performed to evaluate the influence of the PTS (+8°, +4°, Native, −4°, −8°) as well as the anterior–posterior tibial translation in different PTS models. The different models were then assembled and merged with each other (Figure 1).

#### 2.2.1. Preparation for PCL Anatomical Footprints, Bone Tunnels, and PCL Grafts

Anatomical PCL reconstruction provides superior postoperative stability to the posterior translation of the knee joint [28]. By rotating the knee model in SolidWorks, the true lateral and anteroposterior (AP) views of the knee were acquired, as shown in the study by Johannsen et al. [29]. The anatomical footprints of the anterolateral bundle (ALB) and posteromedial bundle (PMB) were obtained using the projection function of SolidWorks software. In the present study, a 10-mm cylinder was used to reconstruct a single-bundle PCL (SB-PCL) graft. A PCL graft with a 10-mm diameter might cover 80% of the tibia and 50% of the femoral footprint [30]. Cylinders with an 11-mm diameter were used for the bone tunnel [31].

In the present study, the tibial tunnel was constructed using the anteromedial approach, and the tibial anteromedial approach was 2.0 cm from the horizontal spacing of the tibial tubercle in the AP view [32]. The midline of the tibial tunnel was determined by joining the anteromedial approach point to the tibial footprint of the PCL graft. The intersection angle between the tibial tunnel center line and the tibia axis vertical plane was the TTA. With this design, 3 different TTAs (30°, 45°, and 60°) were used. As described in previous studies, the femoral tunnel was positioned perpendicular to the axis of the femur on the coronal plane and at a 15° angle to the horizontal line of the medial femoral condyle on the femoral horizontal plane [33]. Considering that a quadruple semitendinosus weave would be used as a graft, and to ensure that the graft was properly placed into the bone tunnel so that it could be fixed well, 15 mm of the PLC graft was inserted into each bone tunnel [17] (Figure 1).

#### 2.2.2. Preparation for aOW-HTO

To evaluate the influence of the PTS on the stress in the “killer turn” area, aOW-HTO was simulated. aOW-HTO was performed according to the procedure reported by previous studies [22,26]. The CAD software program was utilized to determine the starting point of the osteotomy 4 cm distal to the joint line above the tibial tuberosity and to identify the plane perpendicular to the tibial mid-axis as the anterior high tibial osteotomy plane. In the present study, four PTS models were developed based on the PTS angles from a previous study [20,34]. An increase in the PTS (+4°, +8°) was achieved using anterior open-wedge osteotomy, and a decrease in the PTS (−4°, −8°) was achieved using anterior closed-wedge osteotomy. Following aOW-HTO, the only distal tibial model was displaced in the AP direction, with the posterior tibial cortex always retaining continuity in the lateral view.

The four different types of tibial models following aOW-HTO were compared to the original tibial models and, from translation of the tibia, they were measured and recorded. Using SolidWorks, the horizontal displacement distance was determined between the axis of the tibia and the most posterior contours of the medial and lateral femoral condyles in the lateral view [35]. The anterior tibial translation (ATT) for each PTS corresponds to the difference between the tibial displacement distance of this model and the tibial displacement distance of the native model (+8°: 4.46 mm; +4°: 2.27 mm; −4°: −2.39 mm; −8°: −4.13 mm). We found that the anterior tibial translation distances for the +4° and +8° models were consistent with the results of the cadaveric specimens evaluating ATT distances, as in previous studies [22,26]. Furthermore, the ATT of the PTS −4° and −8° models was similar to the results of other studies [36]. We built five PTS models (PTS of +8°, +4°, Native, −4°, −8°) and combined each of the different TTAs with the PTSs’ models using the CAD software program (Figure 1).

### 2.3. Material Properties

After creating each model using SolidWorks, they were imported into the 3-Matic software program (Materialise), where model meshing was performed. The bone model was smoothed once again to confirm that the bone tunnel exits were smooth and that model calculation did not reveal any stress singularities or concentrations. The model was then saved as a body mesh in the C3D10 format. The developed models were exported as Container Database (CDB) files and imported into the ANSYS workbench (Ver. 2021R2, ANSYS, Canonsburg, PA, USA) for an FEA. The model was then built, and material attributes were assigned according to those documented in the scientific literature. The femur and tibia reported linear isotropic elasticity with a Young’s modulus of 7300 MPa and a Poisson’s ratio of 0.3 [37].

In the present study, the material properties of the graft simulated the woven quadruple semitendinosus (QST) [9]. The QST graft is regarded as a fiber-reinforced material, and its constitutive model has been identified as a nonlinear incompressible hyper-elastic material with transverse isotropy. The strain energy ψ function formula of the hyper-elastic material in the present study was as follows:

$$\psi =\psi_{iso}+\psi_{aniso}+\psi_{v}$$

The matrix of the graft was considered to be a hyper-elastic, neo-Hookean material [38,39], and the strain energy function was defined as $\psi_{iso}=C_{1}\left({\bar{I}}_{1}-3\right)$, where $C_{1}$ is the material constant and ${\bar{I}}_{1}$ is the first invariant of the right Cauchy–Green tensor. The collagen fibers of the graft were modeled to support only tensile tension, and the nonlinear relationship between the tensile force and the tensile strain of the fibers was expressed as follows:

$$\lambda \frac{\partial \psi_{aniso}}{\partial \lambda }=\{\begin{array}{c} \;0\;,\lambda <1\\ C_{3\left(e^{C_{4}\left(\lambda -1\right)}-1\right)\;,\;1\leq \lambda \leq \lambda^{*}}\\ \;C_{5}\lambda +C_{6\;},\;\lambda >\lambda^{*} \end{array}$$

where $C_{3}$, *C*_4_, $C_{5}$, and $\lambda^{*}$ are material constants, and the incompressible volume of the graft is expressed as $\psi_{v}=\frac{1}{D}{\left[\ln\left(J\right)\right]}^{2}$. The reciprocal of the bulk modulus is at the D position. J is the jacobian of the movement and *F* denotes the deformation gradient, J=detF. The material order and reciprocal bulk modulus of our graft QST were as follows: $C_{1}$ = 2.75 MPa, $C_{3}$ = 0.065 MPa, $C_{4}$ = 115.89 MPa, $C_{5}$ = 512.73 MPa, $\lambda^{*}$ = 1.042 MPa, and D = 0.00484 MPa^−1^ [40].

### 2.4. Boundary Conditions and Loads

Because of the complex anatomy of the knee joint, the models were simplified by retaining only the tibia, femur, and PCL graft. We fixed the upper ends of the femoral, tibial, and PCL grafts. The distal tibial end of the PCL graft was loaded with 100 N in the direction of the bone tunnel [41] (Figure 1). It was determined that there was frictional sliding between the bone tunnel and graft with a coefficient of friction of 0.3 [42].

Mesh convergence was tested with an element edge length from 1.0 to 0.3 mm using the ligament graft and the bone tunnel exits in this model, while other parts of the bone models kept the surface mesh size at 1.0 mm. With an element edge length from 1.0 to 0.35 mm, the difference in the allowable change of the obtained results was approximately 23.19%. With an element edge length from 0.35 to 0.3 mm, the difference in the allowable change of the obtained results was 0.66%. It was estimated that a further reduction in the element edge length would result in an even smaller difference. Therefore, the element edge length of the ligament graft and the bone tunnel exits in this model were set as 0.3 mm. The total numbers of average nodes and elements were 813,433 and 475,648, respectively.

After performing an FEA on each of the 15 models, the study collected data on the Von Mises stress (VMS) of the grafts. The cancellous bone was more widely distributed than the thin cortex at the proximal tibia [43], and the yield strength of cancellous bone is 5–10 MPa [44]. Therefore, this study defined the area of tibial tunnel contact stress greater than 10 MPa as a “high-contact stress area” and took measurements to record this area.

## 3. Results

### 3.1. Equivalent VMS in the PCL Graft

**Figure 2:**
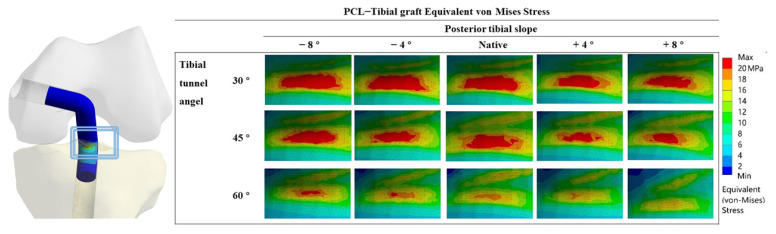
PCL graft equivalent Von Mises Stress distribution around the tibial tunnel entrance after PCL reconstruction.

**Figure 3:**
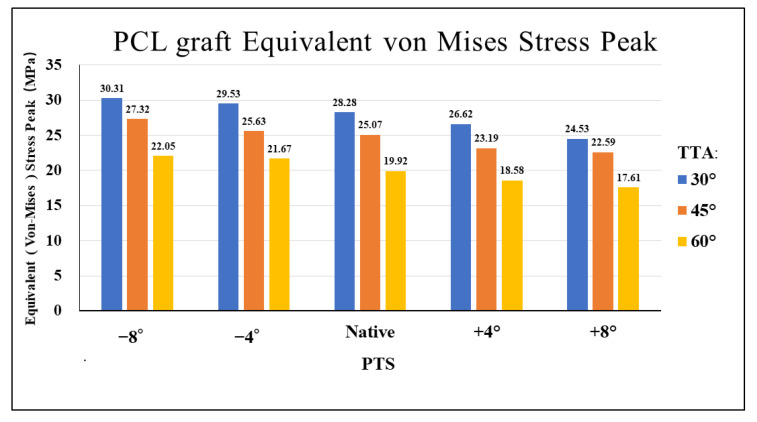
Bar chart of equivalents (Von Mises) stress peak diagrams for different TTAs and PTSs at the entrance of the tibial tunnel. TTA: tibial tunnel angle, PTS: posterior tibial slope.

Figure 2 shows the VMS in the PCL graft at the “killer turn”. Figure 3 shows the peak of the VMS in the PCL grafts in each model. In the same PTS model, the peak of the VMS in PCL grafts decreased as the TTA increased. In the same TTA model, the peak of the VMS in PCL grafts decreased as the PTS increased.

### 3.2. Contact Stress in “Killer Turn”

**Figure 4:**
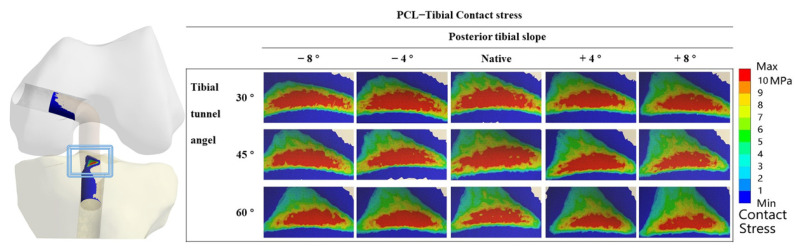
Contact stress distribution around the tibial tunnel entrance following PCL reconstruction.

**Figure 5:**
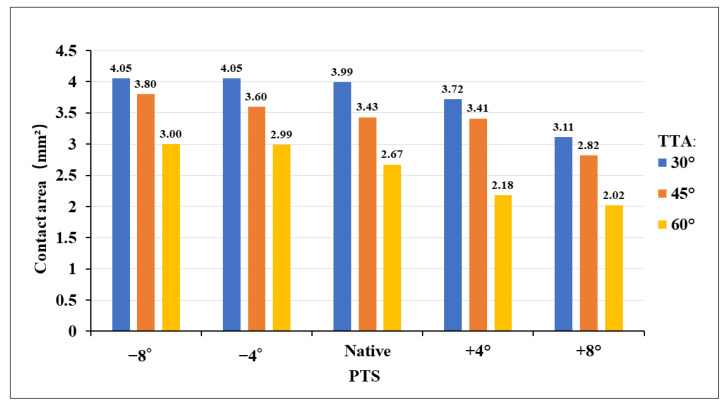
Bar chart contact stress area (greater than 10 MPa) diagram for different TTAs and PTSs at the entrance of the tibial tunnel. TTA: tibial tunnel angle, PTS: posterior tibial slope.

Figure 4 shows the contact stress at the “killer turn” in each model. The results of the “high-contact stress area” (contact area of the contact stress greater than 10 MPa) are shown in Figure 5. The “high-contact stress area” decreased when the PTS angle increased in the same TTA model, and the “high-contact stress area” decreased when the TTA increased in the same PTS model.

## 4. Discussion

The most important finding of this study was that a large TTA during PCL reconstruction weakened the “killer turn” effect. In addition, the study findings suggested that using aOW-HTO simultaneously to create a greater PTS may also be an effective surgical strategy for reducing the “killer turn” effect during PCL reconstruction. The surgical strategy that creates a tibial tunnel using a large TTA combined with the application of aOW-HTO was also related with a reduced area of the high stress in the tibial tunnel inlet at the “killer turn”.

### 4.1. Different TTAs and PTSs during PCL Reconstruction

In the present study, a large TTA reduced the peak VMS in the PCL graft at the “killer turn” area. In the same PTS model, a 60° TTA demonstrated an average decrease in the peak VMS by 19.44% in comparison to that of a 45° TTA. A 60° TTA demonstrated an average decrease in the peak of VMS by 28.37% in comparison to that of a 30° TTA (Figure 3). The reduced peak of the VMS in the PCL grafts may indicate that a large TTA can weaken the “killer turn” effect of the tibial tunnel. This was similar to the results of previous studies that investigated the influence of TTA on PCL reconstruction [13]. However, orthopedic surgeons must safely create the tibial tunnel during PCL reconstruction. According to a study by Mortazavi et al. [1], the TTA should be carefully determined because a thin posterior wall of the tibial tunnel can cause the tunnel wall to fracture during or after PCL reconstruction. Therefore, when creating the tibial tunnel, the distance from the center of the tibial tunnel to the champagne-glass drop-off (CGD) of the posterior tibial cortex must be at least 7 mm [17].

The present study created knee models with different PTSs by simulating aOW-HTO. According to the study findings, a larger PTS reduced the “killer turn” effect in the PCL grafts. For the same TTA, the −4° and −8°PTS models increased the peak VMS in the PCL grafts by 5.1% and 8.9%, respectively, compared to the native PTS model. In contrast, the peak VMS in the PCL grafts in the +4° and +8°PTS models showed an average decrease of 6.7% and 11.6%, respectively, compared to the native PTS model (Figure 3). In a postoperative follow-up study, patients with PCL graft failure had a significantly lower PTS than patients without graft failure, showing a 1.3-fold increase in the odds of graft failure for each one-degree reduction in the PTS [45]. Furthermore, several studies have shown that the PTS has a substantial effect on the in situ forces of the PCL graft [20,46]. Bernhardson et al. found that the in situ force of the PCL increased when the PTS was less than 6°, resulting in an increased incidence of PCL injury [47]. In addition, Shelbourne et al. found that a one-degree increase in the PTS decreased the in situ stress of the PCL by 6 N [46].

Increased attention has recently been paid to the effect of the PTS on PCL reconstruction [2,21,22]. From a biomechanics perspective, the application of aOW-HTO during PCL reconstruction may help improve postoperative clinical outcomes. However, there is insufficient evidence concerning the efficacy and safety of aOW-HTO during PCL reconstruction. aOW-HTO is an inherently invasive surgical procedure, which would increase the possibility of intra-articular infection and delayed union or nonunion after surgery [48]. Therefore, the efficacy and safety of aOW-HTO during PCL reconstruction need to be evaluated in future studies.

Furthermore, this is the first FEA study to propose the use of aOW-HTO to create a larger PTS combined with a large TTA to attenuate the “killer turn” effect during PCL reconstruction. Our results showed that the peak of the VMS in the PCL grafts in the model with a +8° PTS and 60° TTA was much lower than that in the model with a −8° PTS and 30° TTA (Figure 3). The combination of aOW-HTO and the creation of a large TTA during PCL reconstruction may be associated with perioperative complications such as knee joint infection and postoperative delayed recovery [48]. Further studies will be required to validate the safety and usefulness of this combined procedure in a clinical setting.

### 4.2. High-Contact Stress Area

The PCL graft and tunnel are subjected to substantial mechanical stress at the “killer turn” area [49]. Several researchers have reported that the “killer turn” area may contribute not only to graft wear but also to tunnel volume enlargement (TVE) [12,50], and that this may be a major cause of failure of PCL reconstruction [16]. In the present study, the tibial tunnel was created at the anatomical tibial footprint of the PCL. The cancellous bone is reportedly more widely distributed than the thin cortex at the proximal tibia [43]. In some animal studies, the amount of cancellous bone around the ACL graft was found to significantly influence graft-to-bone healing [51]. Therefore, in the present study, a “high-contact stress area” (contact stress greater than 10 MPa) was evaluated at the “killer turn” area of the tibial tunnel. According to the study findings, the “high-contact stress area” decreased when the TTA and PTS increased. Kwon et al. reported that the mean femoral and tibial tunnel enlargements were 10.1–13.8% and 9.9–10.1%, respectively, 1 year after PCL reconstruction [50]. TVE can cause posterior knee laxity after PCL reconstruction [52]. Persistent posterior knee laxity is a factor that can lead to knee osteoarthritis [53]. 

### 4.3. Limitations

Several limitations associated with the present study warrant mentioning. First, the surgical method in our model was created utilizing the CAD software program, therefore the findings may be different from those obtained in actual clinical practice. Second, the study specified linear, isotropic, and homogeneous material properties to simplify the knee model. Therefore, other potential factors that may affect knee stability after PCL reconstruction were not considered. Third, in the present study, we only evaluated how the different TTAs and PTSs affected the “killer turn” effect when the knee flexion angle was 90°. Therefore, the influence of the PTS and TTA on the “killer turn” area in different knee flexion angles was not assessed. Finally, the present study was conducted using data from a single healthy subject. The subject’s specific knee morphologies were likely to affect the results.

## 5. Conclusions

The study findings suggested that using aOW-HTO to increase the PTS as well as creating a large TTA may effectively weaken the “killer turn” effect during PCL reconstruction. Compared to 30° TTA, 60° TTA decreased the VMS of the PCL graft at the “killer turn” by 28.37% on average. In comparison to −8°PTS, +8°PTS reduced the VMS of the PCL graft at the “killer turn” by 23.35% on average. Additionally, an increased PTS and TTA decreased the high-contact stress area at the tibial tunnel inlet, which will be related to the TVE following PCL reconstruction. The usefulness and safety of this surgical strategy (combination of aOW-HTO and a larger TTA) to attenuate the “killer turn” effect will need to be evaluated in future clinical studies.

## Data Availability

The data presented in the present study are available on request from the corresponding author.
